# Supplementary material for: Mystery(n) Phenotypic Presentation in Europeans: Report of Three Further Novel Missense RNF213 Variants Leading to Severe Syndromic Forms of Moyamoya Angiopathy and Literature Review
Source: Int J Mol Sci. 2022 Aug 11;23(16):8952. doi: 10.3390/ijms23168952 (PMC9408709; doi:10.3390/ijms23168952)
Supplement: Supplementary file 1 [file ijms-23-08952-s001.zip › Figure S1.pdf]

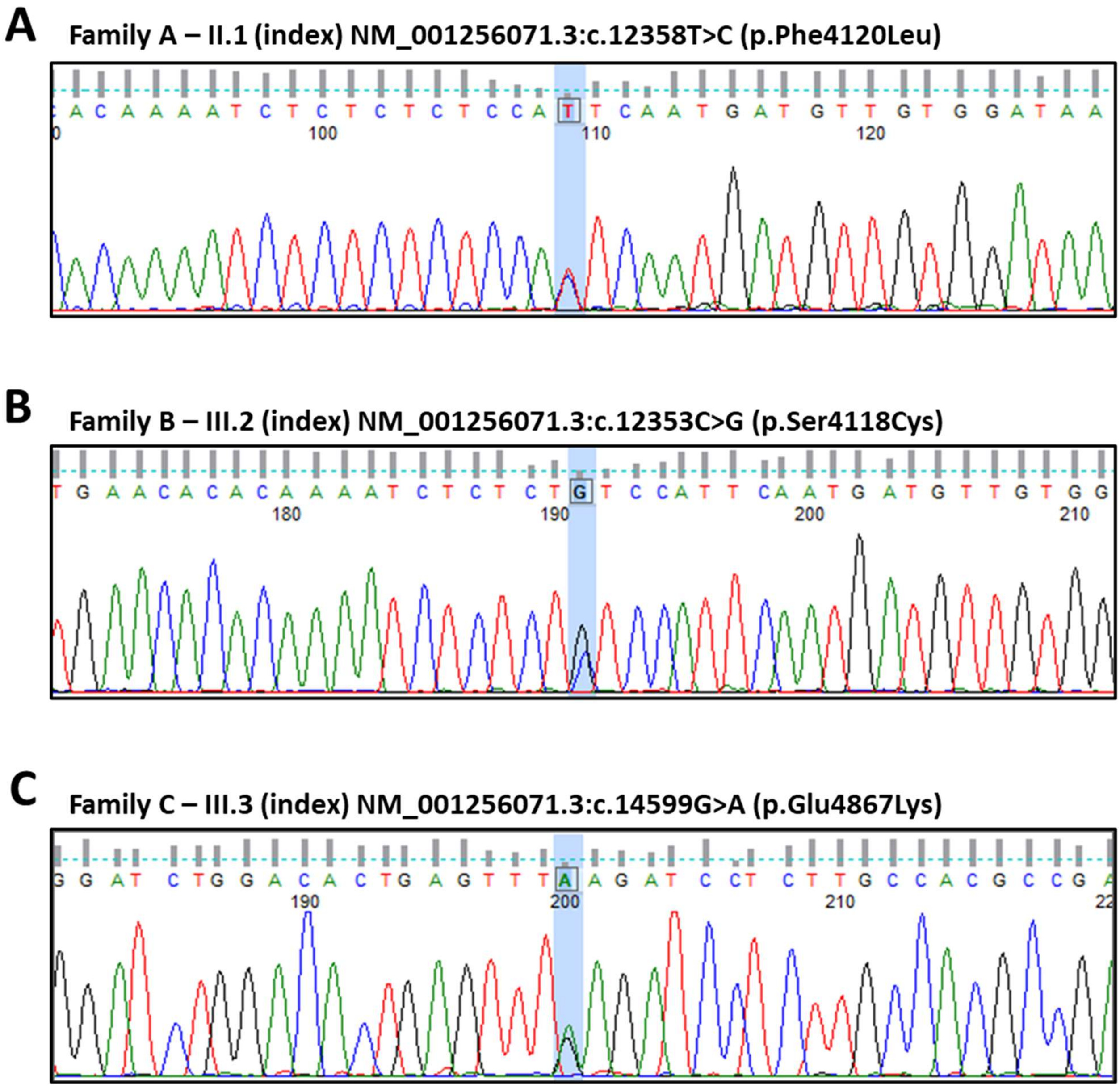

Figure S1. *Electropherogram of the RNF213 variants identified in probands from families A-C.* Sanger sequencing of the specific *RNF213* exon for each index cases from families A-C. The identified heterozygous missense variants are highlighted with a light blue background.
