# Supplementary material for: Mystery(n) Phenotypic Presentation in Europeans: Report of Three Further Novel Missense RNF213 Variants Leading to Severe Syndromic Forms of Moyamoya Angiopathy and Literature Review
Source: Int J Mol Sci. 2022 Aug 11;23(16):8952. doi: 10.3390/ijms23168952 (PMC9408709; doi:10.3390/ijms23168952)
Supplement: Supplementary file 1 [file ijms-23-08952-s001.zip › Table S1.pdf]

Table S1: *Demographical, clinical, and radiological features of European patients already reported in literature.*

Reference	Patient ID[1]	Proband Relative	Sex	Mutation	MMA/other cerebrovascular condition/ bilateral or unilateral	Age at onset (yrs)	MMA symptoms	Atypical angiographic features	HBP	Extracerebral arterial or cardiac anomalies	Hypertransaminasemia/Hepatitis	Other systemic features
[13]	MM004 n.r.IIn.r.10	P	M	p.Arg4019Cys	MMA/B	3	n.r.	n.r.	n.r.	n.r.	n.r.	n.r.
	MM096_IVn.r.4	P	M	p.Asp4013Asn	MMA/B	30	n.r.	n.r.	n.r.	n.r.	n.r.	n.r.
	MM096_Vn.r.1	R	M		MMA/B	8	n.r.	n.r.	n.r.	n.r.	n.r.	n.r.
	MM096_IIIn.r.5	R	M		No	56	n.r.	n.r.	n.r.	CAD	n.r.	n.r.
	MM096_IIIn.r.4	R	F		MMA/n.r.	30	n.r.	n.r.	n.r.	n.r.	n.r.	n.r.
	MM0011_IVn.r.11	P	F	P.Glu4950_Phe4951Ins7	MMA/B	5	n.r.	n.r.	n.r.	n.r.	n.r.	n.r.
	MM060_IIn.r.1	P	F	p.Val5163Ile	MMA/B	8	n.r.	n.r.	n.r.	n.r.	n.r.	n.r.
	MM006_IIn.r.1	P	M	p.Arg3922Gln	MMA/B	5	n.r.	n.r.	n.r.	n.r.	n.r.	n.r.
	MM006_IIn.r.6	R	F		MMA/B	2.5	n.r.	n.r.	n.r.	n.r.	n.r.	n.r.
	MM044_IIn.r.8	P	M	p.Arg4019Cys	MMA/B	6	n.r.	n.r.	n.r.	n.r.	n.r.	n.r.
	MM042_IIn.r.10	P	F	p.Ala529del	MMA/B	50	n.r.	n.r.	n.r.	n.r.	n.r.	n.r.
	MM131_IIn.r.11	P	M	p.Lys4115del	MMA/B	0.75	n.r.	n.r.	n.r.	n.r.	n.r.	n.r.
[22]	Proband	P	F	p.Ser4118Phe	MMD/B	0.25	Acute onset of focal seizures	Involvement of posterior cerebral and superior cerebellar arteries	n.r.	Marked narrowing of bilateral internal carotid arteries; mild stenosis of infrarenal abdominal aorta	Elevated transaminases; abnormal liver biopsy	None
[8,30]	Patient 1	P	M	p.Asp4013Asn	MMA/B	44	CI	n.r.	n.r.	n.r.	n.r.	n.r.
[30]	IIn.r.2 fam 1	P	F	p.Val4146Ala	MMA/B	21	TIA	n.r.	No	Thickening of the carotid bulb in both internal carotid arteries, prolapse of mitral valve	No	None
	IIn.r.1 FAM 2	P	F	p.Arg4019Cys; p.Glu4042Lys	MMA/B	28	CI	n.r.	n.r.	n.r.	n.r.	n.r.
	IIIn.r.2 FAM 3	P	F	p.Trp4677Leu	MMA/B	27	Paresis	n.r.	Yes	None	n.r.	n.r.
	IIn.r.2 FAM 3	R	F		MMA/U	48	CI	n.r.	Yes	Severe stenosis in the renal arteries	n.r.	n.r.
[16]	M001_6	P	F	p.Ala1135Val p.Cys4032Arg	MMA/B	13	TIA, headache, movement disorders	Vertebrobasilar impairment	No	No	n.r.	No
	M002_3	P	M	p.Arg4062Gln	MMA/B	5	CI, TIA, headache	None	Yes	No	n.r.	No
	M003_4	P	M	p.Asp4122Val	MMA/B	5	TIA, headache	Posterior cerebral artery occlusion	Yes	Severe pulmonary arterial hypertension	Regressive unresolved hepatitis at 14 months	No
	M004_3	P	M	p.Thr1705Lys; p.H4014Asn	MMA/B	3	CI, TIA, headache	None	No	No	n.r.	No
	M005_5	P	F	p.Pro1721Leu	MMA/U	22	Headache	None	No	n.r.	n.r.	Left morning glory syndrome, pituitar insufficiency, meningoencephalocele, choanal malformation
	M006_3	P	F	p.Val3933Met	MMA/B	20	CI	n.r.	Yes	n.r.	n.r.	n.r.
	M006_5	R	F		MMA/B	28	CI, headache	None	Yes	No	n.r.	No
	M007_4	P	M	p.Asp4013Asn	MMA/B	3	CI, headache	None	n.r.	No	n.r.	No
	M007_5	R	F		MMA/B	1.5	CI, TIA, epilepsy	None	n.r.	No	n.r.	No
	M008_4	P	F	p.Arg4019Cys; p.Glu4042Lys	MMA/B	40	CI	None	Yes	No	n.r.	Right thumb malformation, facial dysmorphism, short stature
[16,17]	M009_3	P	F	p.P395Leu	MMA/B	<14	TIA, movement disorder, headache	None	No	No	n.r.	No
	M010_10	R	F	p.P4033Leu	MMA/B	10	CI, TIA, headache	None	No	No	n.r.	No
	M010_5	P	F		MMA/B	41	CI, TIA	None	n.r.	No	n.r.	Livedo racemosa
	M011_4	P	F	p.Cys118Arg;p.His4051Pro	MMA/B	3	CI	None	n.r.	n.r.	n.r.	Overweight, recurrent otitis
	M012_3	P	M	p.Leu133Met; p.Ile209Asn	MMA/B	29	CI, TIA, ICH, headache	None	Yes	No	n.r.	No
	M013_4	P	F	p.Ala1844Thr	MMA/B	40	CI	None	Yes	No	n.r.	No
	M014_3	P	F	p.Arg3846His	MMA/B	33	CI, TIA	None	No	No	n.r.	Type 1 diabetes
[16]	M015_7	P	M	p.Ala4399Thr	MMA/B	49	CI	None	No	No	n.r.	Lentigo maligna melanoma

Table S1: *Demographical, clinical, and radiological features of European patients already reported in literature.*

Reference	Patient ID[1]	Proband Relative	Sex	Mutation	MMA/other cerebrovascular condition/ bilateral or unilateral	Age at onset (yrs)	MMA symptoms	Atypical angiographic features	HBP	Extracerebral arterial or cardiac anomalies	Hypertransaminasemia/Hepatitis	Other systemic features
[16,17]	M016_3	P	F	p.Pro4608Ser	MMA/B	37	CI, headache, epilepsy	None	No	No	n.r.	No
	M017_4	P	F	p.Gly4640Ser	MMA/B	18	ICH	None	Yes	n.r.	n.r.	No
	M018_3	P	F	p.Ala4399Thr	MMA/B	36	CI, TIA	Stenosis of the distal MCA M1 segment without neovessels on the right side; ostial vertebral stenosis	Yes	No	n.r.	No
	M019_7	P	M	p.Ala3927Thr	MMA/B	27	CI, epilepsy	Posterior cerebral arteries	No	n.r.	n.r.	No
	M019_3	R	F		MMA/B	55	CI	Vertebrobasilar impairment	Yes	No	n.r.	Hypothyroidism
	M019_5	R	M		Stenosis of the left ACA A1 segment without neovessels	59	n.r.	n.r.	Yes	n.r.	n.r.	Varicosis
[18]	IIIn.r.9	P	F	p.Lys4185Glu	MMA/B	4	Left-sided weakness and aphasia brought on by excitement, persistant headache	n.r.	n.r.	n.r.	n.r.	n.r.
	IIIn.r.1	R	M		MMA/n.r.	5	Stroke	n.r.	n.r.	n.r.	n.r.	n.r.
	IIIn.r.2	R	F		MMA/n.r.	n.r.	Movement disorder	n.r.	n.r.	n.r.	n.r.	n.r.
	IIn.r.7	R	F		MMA/n.r.	21	Stroke	n.r.	n.r.	n.r.	n.r.	n.r.
[21]	Patient 1	P	F	p.Thr4114Arg	MMA/B	0.8	Status epilepticus with decreased movement in the left upper limb	None	No	Arterial vessels throughout the cerebrovascular circulation and the femoral arteries were small in caliber, femoral arteries laterally located	n.r.	n.r.
	Patient 2, MM194	P	M	p.Pro3996_Cys3997 delinsGlyLeuGly	MMA/B	0.6	Seizures	None	Yes	Bilateral renal artery stenosis, iliac artery occlusion	Levetiracetam was initiated, elevated transaminases resolved with transition to oxcarbazepine	Hypertensive dilated cardiomyopathy, antiphospholipid antibody syndrome
	Patient 3, MM169	P	M	p.Phe4120Leu	MMA/B	1.25	Right-sided paralysis and seizures	None	Yes	Bilateral renal artery stenosis, aorto–left renal artery bypass due to stenosis at 23 yrs	n.r.	Hypothyroidism
[8]	n.r.	P	n.r.	p.Asn3962Asp	MMA/n.r.	n.r.	n.r.	n.r.	n.r.	n.r.	n.r.	n.r.
	CAU_ped1_12	P	n.r.	p.Asp4013Asn	Mild ischemic stroke at the age of 30		n.r.	n.r.	n.r.	n.r.	n.r.	n.r.
	CAU_Ped1_122	R	n.r.		MMA/n.r.	5	n.r.	n.r.	n.r.	n.r.	n.r.	n.r.
	CAU_Ped1_123	R	n.r.		MMA/n.r.	9	Symptoms of involuntary movement	n.r.	n.r.	n.r.	n.r.	n.r.
	CAU_Ped1_124	R	n.r.		MMA/n.r.	3	Ischemic stroke	n.r.	n.r.	n.r.	n.r.	n.r.
	n.r.	P	n.r.	p.Arg4062Gln	MMA/n.r.	n.r.	n.r.	n.r.	n.r.	n.r.	n.r.	n.r.
	n.r.	P	n.r.	p.Pro4608Ser	MMA/n.r.	n.r.	n.r.	n.r.	n.r.	n.r.	n.r.	n.r.

[1]As reported in the published article (see reference). Abbreviations: B = bilateral; U = unilateral; CAD = premature coronary artery disease; CI = cerebral infarct; F = female; HBP = high blood pressure; ICA = internal carotid artery; ICAO = internal carotid artery occlusion; ICH = intracerebral brain hemorrhage; ID = patient identifier; L = left; M = male; MCA = middle cerebral artery; MMA = moyamoya angiopathy; MMD = moyamoya disease; TIA = transient ischemic attack; n.a. = not available; n.r. = not reported.
