# Supplementary material for: Mystery(n) Phenotypic Presentation in Europeans: Report of Three Further Novel Missense RNF213 Variants Leading to Severe Syndromic Forms of Moyamoya Angiopathy and Literature Review
Source: Int J Mol Sci. 2022 Aug 11;23(16):8952. doi: 10.3390/ijms23168952 (PMC9408709; doi:10.3390/ijms23168952)
Supplement: Supplementary file 1 [file ijms-23-08952-s001.zip › Table S2.pdf]

Table S2: *Selected MMA genes from the cerebral vascular malformations (Version 2.59) panel.*

Gene Symbol	Model of Inheritance	Phenotypes	OMIM
ACTA2	Monoallelic	Moyamoya disease 5	102620
GUCY1A3	Biallelic	Moyamoya 6 with achalasia	139396
RNF213	Both	{Moyamoya disease 2, susceptibility to}	613768
SAMHD1	Biallelic	Moyamoya disease	606754
SLC2A10	Biallelic	Arterial tortuosity syndrome;Moyamoya disease	606145
CBL	Monoallelic	Early-onset moyamoya angiopathy;Noonan syndrome-like disorder with or without juvenile myelomonocytic leukemia	165360
MYH11	Monoallelic	Moyamoya-like angiopath;Aortic aneurysm, familial thoracic 4	160745
NF1	Monoallelic	Moyamoya disease;Neurofibromatosis, type 1	613113
PCNT	Biallelic	Moyamoya disease;Microcephalic osteodysplastic primordial dwarfism, type II	605925
ATP7A	X-Linked	Moyamoya disease	300011
BRCC3	X-Linked	Moyamoya disease	300617
ELN	Monoallelic	Moyamoya disease;Aneurysm, intracranial berry, 1	130160
JAG1	Monoallelic	Moyamoya disease;Alagille syndrome 1,	601920
NOTCH3	Monoallelic	Cerebral Autosomal Dominant Arteriopathy with Subcortical Infarcts and Leukoencephalopathy (CADASIL);Moyamoya disease	600276
CCER2	Monoallelic	Moyamoya disease	617634
MRVI1	Monoallelic	{Moyamoya angiopathy, susceptibility to}	604673
PALD1	Monoallelic	{Moyamoya angiopathy, susceptibility to}	614656
