# Supplementary material for: Mystery(n) Phenotypic Presentation in Europeans: Report of Three Further Novel Missense RNF213 Variants Leading to Severe Syndromic Forms of Moyamoya Angiopathy and Literature Review
Source: Int J Mol Sci. 2022 Aug 11;23(16):8952. doi: 10.3390/ijms23168952 (PMC9408709; doi:10.3390/ijms23168952)
Supplement: Supplementary file 1 [file ijms-23-08952-s001.zip › Table S3.pdf]

Table S3: *Distribution of variants reported in literature along RNF213 and in silico evaluation of their pathogenicity.*

Mutation	Ethnicity	Domain	PROVEAN		Polyphen2		MutPred2	MAF (gnomAD)	PMID references[1]
			Score	*Prediction (cutoff = -2.5)*	*Score*	*Prediction*	*Score (cutoff = 0.5)*		
A529del	European	N-arm (stalk)	-5.648	Deleterious	n.a.	n.a.	n.a.	0.000000	25278557
A1622V	Asian		-2.04	Neutral	0.220	Benign	0.208	0.000171	25956231
M3891V	Asian		-1.694	Neutral	0.007	Benign	0.129	0.000000	21048783
R3922Q	European	E3 (back)	-2.667	Deleterious	0.992	Probably Damaging	0.296	0.000032	25278557
A3927T	European		-2.178	Neutral	0.990	Probably Damaging	0.416	0.000004	30908154
V3933M	Asian		-1.883	Neutral	0.998	Probably Damaging	0.696	0.000004	25956231
N3962D	European		-1.289	Neutral	0.012	Benign	0.118	0.000050	21799892
P3996_C3997delinsGLG	European	RING	-19.317	Deleterious	n.a.	n.a.	n.a.	0.000000	33568546
C3997Y	European		-8.783	Deleterious	1.000	Probably Damaging	0.886	0.000000	25278557
P4007R	Asian		-6.883	Deleterious	1.000	Probably Damaging	0.816	0.000000	23110205
D4013N	European		-2.35	Neutral	0.909	Possibly Damaging	0.577	0.000008	21799892, 25278557
R4019C	European		-3.423	Deleterious	0.992	Probably Damaging	0.649	0.000615	25278557
P4033L	European		-8.167	Deleterious	1.000	Probably Damaging	0.666	0.000004	30908154
R4062Q	European		-2.8	Deleterious	0.993	Probably Damaging	0.666	0.000000	21799892
I4076V	Asian		-0.163	Neutral	0.002	Benign	0.212	0.000012	25278557
T4114R	European		-5.133	Deleterious	1.000	Probably Damaging	0.848	0.000000	33568546
K4115del	European		-11.05	Deleterious	n.a.	n.a.	n.a.	0.000000	25278557
S4118F	European		-5.4	Deleterious	1.000	Probably Damaging	0.740	0.000000	26198278
S4118C	European	E3 (shell)	-4.45	Deleterious	1.000	Probably Damaging	0.735	0.000000	This report
F4120L	European		-5.433	Deleterious	1.000	Probably Damaging	0.697	0.000000	33568546, This report
R4131C	Asian		-7.333	Deleterious	1.000	Probably Damaging	0.841	0.000014	25956231
K4185E	European		-2.333	Neutral	0.995	Possibly Damaging	0.492	0.000000	31645973
A4188T	European		-2.081	Neutral	0.962	Probably Damaging	0.164	0.000000	31645973
D4273E	European		-2.7	Deleterious	0.981	Probably Damaging	0.393	0.000000	25278557
Q4367L	Asian		-4.772	Deleterious	0.012	Benign	0.334	0.000000	23110205
V4567M	Asian		-0.595	Neutral	0.190	Benign	0.236	0.000085	21048783
T4586P	Asian		-4	Deleterious	1.000	Probably Damaging	0.845	0.000000	23110205
P4608S	European		-2.758	Deleterious	0.478	Possibly Damaging	0.250	0.000004	21799892
L4631V	Asian		-1.85	Neutral	1.000	Probably Damaging	0.344	0.000000	23110205
K4732T	European		-2.067	Neutral	0.799	Possibly Damaging	0.339	0.000658	25278557
V4765M	Asian	E3 (core)	-1.683	Neutral	0.997	Probably Damaging	0.280	0.000000	21048783
D4863N	Asian		-2.408	Neutral	0.372	Benign	0.355	0.000060	21799892
E4867K	European		-2.2	Neutral	0.909	Possibly Damaging	0.607	0.000000	This report
E4950D	Asian		-1.483	Neutral	0.909	Possibly Damaging	0.339	0.000226	21799892, 23110205
E4950_F4951ins7	European	CTD	n.a.	n.a.	n.a.	n.a.	n.a.	0.000000	25278557
A5021V	Asian		-0.456	Neutral	0.128	Benign	0.181	0.000311	21799892, 23110205
M5136I	Asian		-2.2	Neutral	0.473	Possibly Damaging	0.625	0.000000	23110205
D5160E	Asian		-1.533	Neutral	0.410	Benign	0.240	0.000000	21799892
V5163I	European		-0.375	Neutral	0.919	Possibly Damaging	0.081	0.000152	25278557
E5176G	Asian		-3.892	Deleterious	0.256	Benign	0.308	0.000008	21799892

[1] Unique identifier of published article in PubMed (pubmed.ncbi.nlm.nih.gov). Abbreviations: CTD = C-terminal domain; MAF = minor allele frequency; n.a.= not available.
